# The Impact of Tai Chi Exercise on Self-Efficacy, Social Support, and Empowerment in Heart Failure: Insights from a Qualitative Sub-Study from a Randomized Controlled Trial

**DOI:** 10.1371/journal.pone.0154678

**Published:** 2016-05-13

**Authors:** Gloria Y. Yeh, Caroline W. Chan, Peter M. Wayne, Lisa Conboy

**Affiliations:** 1 Division of General Medicine and Primary Care, Department of Medicine, Beth Israel Deaconess Medical Center, Boston, Massachusetts, United States of America; 2 Osher Center for Integrative Medicine, Harvard Medical School, Boston, Massachusetts, United States of America; 3 Division of Preventive Medicine, Brigham and Women’s Hospital, Boston, Massachusetts, United States of America; 4 New England School of Acupuncture, Boston, Massachusetts, United States of America; University of Bologna, ITALY

## Abstract

**Objective:**

To qualitatively explore perceived physical and psychosocial effects and overall patient experience associated with a 12-week tai chi (TC) intervention and an education group in a clinical trial of patients with chronic heart failure (HF).

**Subjects and Methods:**

We randomized 100 patients with chronic systolic HF (NYHA Class 1–3, ejection fraction≤40%) to a 12-week group TC program or an education control. At 12-weeks, semi-structured interviews were conducted on a random subset (n = 32; n = 17 in TC, n = 15 in control), audiorecorded and transcribed verbatim. Two independent reviewers extracted information using grounded-theory methods for emergent themes. We explored similarities and differences in themes/sub-themes between the groups, and examined qualitative association with changes from baseline to post-intervention in previously reported quantitative measures (e.g., Minnesota Living with HF, Cardiac Exercise Self Efficacy and Profile of Mood States).

**Results:**

The mean age (±SD) of participants was 68±9 years, baseline ejection fraction 29±7%, and median New York Heart Association class 2 HF. We idenitifed themes related to the patient’s experience of illness, perceptions of self, and relationship to others. Specific psychosocial and physical benefits were described. Common themes emerged from both groups including: social support and self-efficacy related to activity/exercise and diet. The tai chi group, however, also exhibited a more global empowerment and perceived control. Additional themes in TC included mindfulness/self-awareness, decreased stress reactivity, and renewed social role. These themes mirrored improvements in previously reported quantitative measures (quality-of-life, self-efficacy, and mood) in TC compared to control. Patients in TC also reported physical benefits (e.g., decreased pain, improved energy, endurance, flexibility).

**Conclusion:**

Positive themes emerged from both groups, although there were qualitative differences in concepts of self-efficacy and perceived control between groups. Those in tai chi reported not only self efficacy and social support, but overall empowerment with additional gains such as internal locus of control, self-awareness and stress management. Future studies of mind-body exercise might further examine perceived control, self-efficacy, and locus-of-control as potential mediators of effect.

## Introduction

Chronic heart failure is a clinical syndrome and the common end pathway of many cardiovascular diseases such as coronary artery disease and hypertension. It affects over 5 milion people in the US, and is the most common hospitalization diagnosis among the Medicare population, costing an estimated $32 billion per year.[1] While pharmaceuticals and medical devices have improved patient lives, many continue to suffer and the natural course of disease is progressively debilitating.

Importantly, heart failure is now recognized as a complex syndrome with impact on multiple dimensions of patients’ lives. Physically, patients suffer from decreased exercise tolerance, progressive dyspnea, deconditioning and fatigue. Mentally and psychosocially, patients describe emotional distress, feelings of powerlessness, helplessness, depression, anxiety, and social role dysfunction.[2] It is well recognized that comprehensive management needs to address these multi-dimensional domains and that maintenance of quality of life is critical. Successful living with heart failure often requires difficult behavior change and life-long adherence to self-care and medical regimens. Recent studies have sought to better understand the role of patient beliefs and how sense of control in their health (perceived control and self-efficacy) can facilitate well-being. For example, while exercise can improve quality-of-life and reduce heart failure-related hospitalizations, initial adoption as well as long term adherence is problematic.[3–6] It has been recognized that exercise self-efficacy is one of the strongest independent predictors of physical activity behavior in HF.[7] Self-efficacy and perceived control may be associated with improved quality of life in patients with cardiovascular illness, asthma and breast cancer.[8–10] These relationships, however, are complex and less well understood, specifically in HF.

There has been emerging interest in mind-body exercise for patients with HF, as it is described as relatively gentle and accessible to even the elderly and more deconditioned. Moreover, purported mechanisms of mind-body therapies may be highly relevant for HF pathophysiology, such as targeting aspects of breathing and relaxation, decreasing sympathetic overdrive, modulating autonomic tone, and addressing the neurohormonal axis.[11–12] Growing in popularity in the United States, tai chi is one such mind-body exercise with origins in Chinese martial arts and healing. Tai chi uses detailed regimens of slow, gentle physical movements integrated with breathing techniques and cognitive tools (eg. somatic awareness, imagery, and meditation).[13] Systematic reviews of tai chi have reported benefits across multiple chronic medical conditions, including musculoskeletal/rheumatological, neurological, and cardiolulmonary disease.[14] We have previously reported significant increases in quality of life and cardiac exercise self-efficacy with tai chi in patients with HF.[15] Other invesigators have also suggested that tai chi may improve self-efficacy in stroke surviviors, Parkinsons disease, and osteopenia.[16–18]

With a growing appreciation for patient-centered outcomes, and recognition that patient experience is just as important as physiological tests and measurements, many studies have utlized mixed methods, combining both quantitative and qualitative data for a more comprehensive assessment. The addition of qualitative methods to trials allows the investigation of more abstract concepts that are difficult to measure and can help to explain unexpected findings, describe participant motivations and beliefs, and provide insight into causal mechanisms underlying complex human behaviors and decision making processes.[19–21] In this context we utilized a qualitative sub-study to expand upon the quantitative clinical trial data. (previously published).[15] We sought to better understand patient experiences, perceived changes, and health benefits associated with a tai chi mind-body exercise program as compared to a HF education control, and to generate new hypotheses regarding benefits of tai chi and potential mediators of effect with respect to health-related quality of life.

## Methods

### Quantitative Parent Study

**Fig 1:**
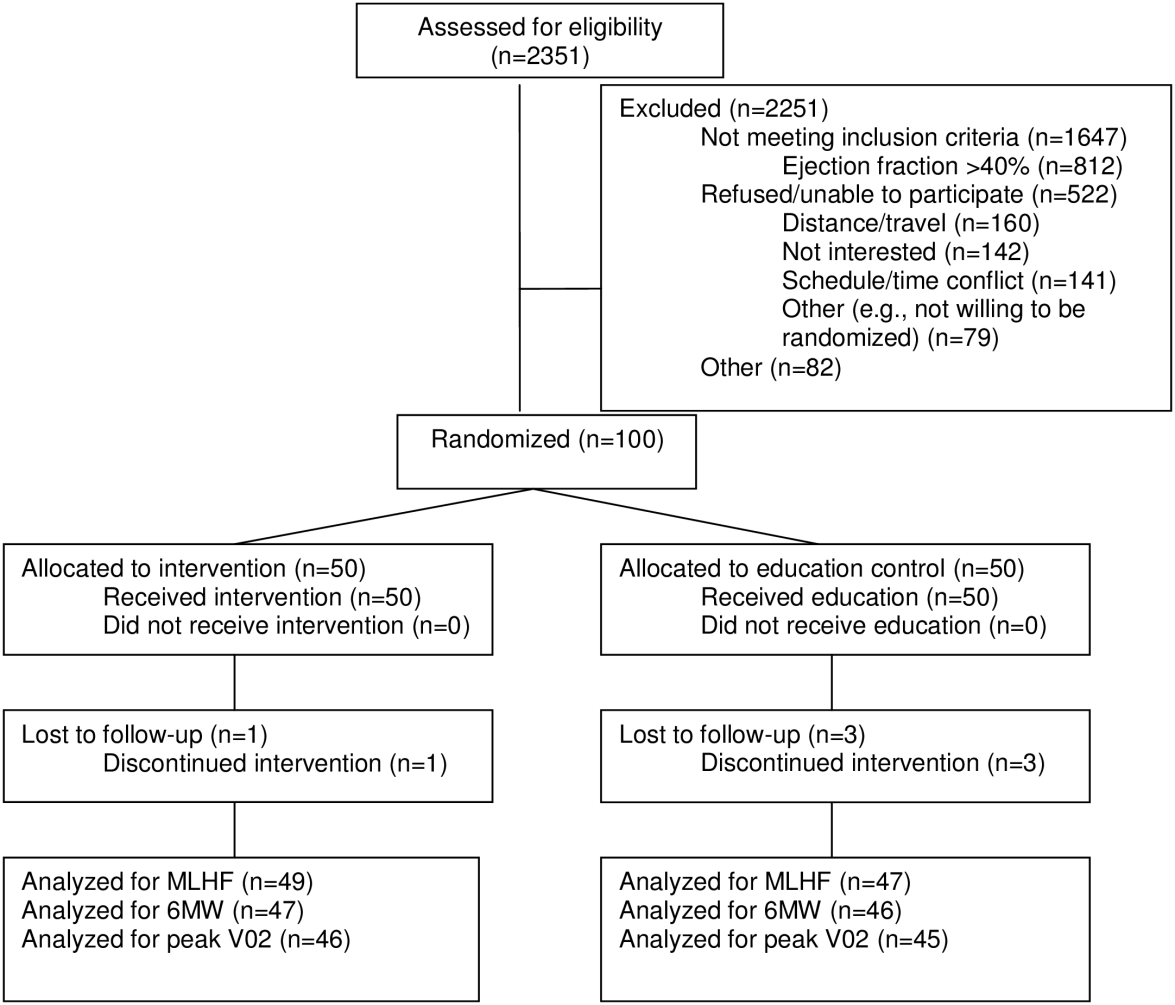
CONSORT Diagram for Parent Study.

Detailed methods are described elsewhere, but a brief summary of the parent study is provided here for context.[15] We recruited 100 patients from ambulatory clinics at three academic medical centers and affiliated practices in and around Boston, MA including Beth Israel Deaconess Medical Center, Brigham and Women’s Hospital, and Massachusetts General Hospital. All participants provided written informed consent. This research was approved by each institution’s human subject review board at Beth Israel Deaconess Medical Center, Partners Healthcare, Harvard Medical School, and Hebrew Senior Life. Patients were randomly assigned via permuted block randomization with variable block size to receive either a 12-week tai chi exercise program or a heart health education program (attention control). All participants continued to receive usual care, which included pharmacologic therapy, and general exercise advice per American College of Cardiology guidelines. Inclusion criteria included: individuals with 1) physician diagnosis of chronic HF, 2) left ventricular ejection fraction ≤40% in the past 2 years, 3) stable medical regimen, 4) New York Heart Association Class I, II, or III. CONSORT diagram is provided in Fig 1.

The tai chi intervention consisted of one-hour group classes held twice weekly for 12 weeks. The protocol included traditional warm-up exercises followed by five simplified tai chi movements. Each cohort was taught by one or two certified and experienced instructors (average experience of 20 years, total number of study instructors = 6).[15] Warm-up exercises included weight shifting, arm swinging, gentle stretches of the neck/shoulders/spine/arms/legs, visualization techniques, and traditional breathing methods. Exercises focused on releasing tension in the physical body, incorporating mindfulness and imagery into movement, increasing awareness of breathing and promoting overall relaxation of body and mind. The core tai chi movements were adapted from Master Cheng Man-Ch’ing’s Yang-style short form and performed repetitively. We provided a 35-minute instructional videotape that outlined the exercises presented in class. Patients were encouraged to practice at home at least three times per week. Class attendance was monitored and compliance with home practice was tracked via self-report logs. Participants also received weekly the same weekly education pamphlets used in our education control (see below).

Patients in the attention control group attended education sessions twice weekly.^15^ Classes were led by a nurse practitioner and followed the content of the 11 Heart Failure Society of America education modules. An additional module on cholesterol was added using patient information from the National Heart, Lung and Blood Institute (NHLBI). The 12 weekly modules (available online at www.hfsa.org) included: 1) Taking control of your HF, 2) How to follow a low-sodium diet, 3) HF medicines, 4) Self-care and dealing with HF symptoms, 5) Exercise and activity, 6) Managing feelings about HF, 7) Tips for family and friends, 8) Lifestyle changes, 9) Advanced directives, 10) Heart rhythm problems, 11) New HF treatments, 12) High blood cholesterol. Each module consisted of a printed pamphlet that was handed out weekly and discussed during the two sessions.

We obtained all measures at baseline and 12 weeks. Questionnaires and functional assessments were also obtained at 6 weeks in the event that 12-week data were unavailable. Quantitative measures included: peak exercise capacity and functional status (cardiopulmonary exercise stress test, six minute walk, timed up and go); health-related quality-of-life (Minnesota Living with HF questionnaire), symptoms,), psychosocial functioning and mood (Cardiac Exercise Self-Efficacy Instrument, Profile of Mood States); and biomarkers (BNP, catecholamines, immune and inflammatory markers). We also tracked physical activity (CHAMPS Physical Activity Questionnaire), medications, healthcare utilization and monitored adverse events.[15]

### Qualtitative Sub-Study

At 12-weeks, semi-structured interviews were conducted on a random subset of the 100 participants (n = 32; n = 17 in tai chi, n = 15 in control). The guiding questions were as follows:1) Tell me about how your heart condition may have impacted your life. 2) Can you tell me what your experience was being in the study group? 3) What are the ways, if any, have you found this program to be helpful? What negative effects, if any, have you experienced or anything that might concern you? 4) What do you feel has been the most important aspect of what you have learned? 5) Do you believe that meditative exercise can affect your mental health? Your physical health?

Interviews were audiorecorded and led by a trained qualitative expert who was not otherwise involved in patient interventions or testing. Recordings were then transcribed verbatim. Two reviewers independently extracted information using grounded-theory methods for emergent themes.[22] Utilizing the process of constant comparison, we identified themes in transcripts through an iterative approach to analysis.[23–24] We first coded and extracted information from a random selection of 5 transcripts in each intervention group. Investigators then discussed emergent themes, created categories and subcategories of themes as a template to code and extract information from a second round of transcripts. The process was repeated, extracting quotes within existing themes and creating new themes/subthemes. This was facilitated by the use of a matrix display of coded and categorized data in cells.[25] All transcripts were coded and we reached thematic saturation. We then explored commonalities as well as differences in themes/sub-themes between the groups and then matched emergent themes with previously reported quantitative measures (e.g., Minnesota Living with HF, Cardiac Exercise Self Efficacy and Profile of Mood States).[15]

To ensure rigor in the process and address potential threats to validity, we employed strategies of random sampling within the larger study, careful documentation of the entire research process (audit trail), utilizing an interviewer trained and experienced in qualitative analysis, using multple coders and a multi-disciplinary research team, and use of investigative triangulation with two authors comparing coding decisions and a third arbiter as necessary.[26–27]

## Results

**Fig 2:**
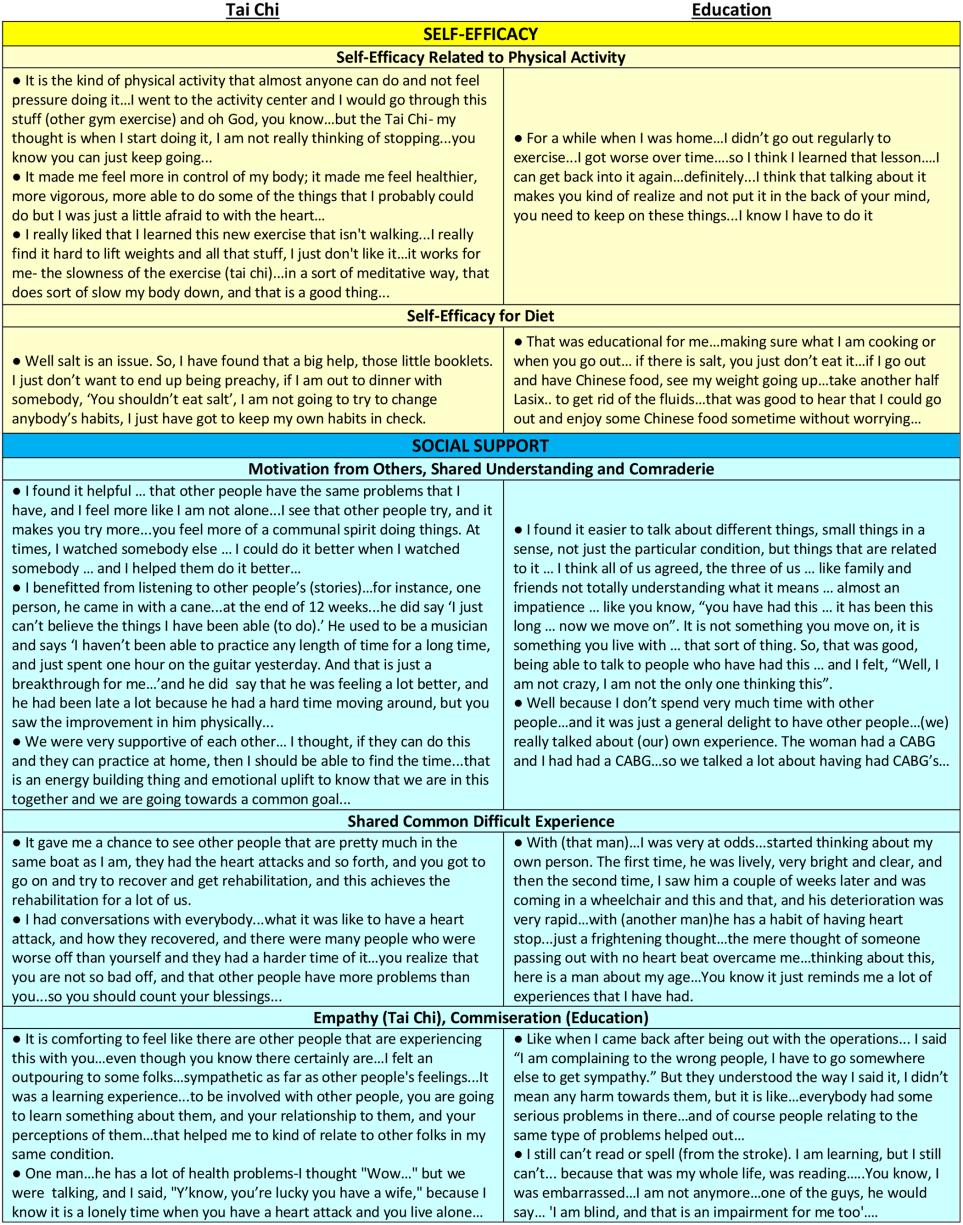
Representative Quotes Within Emergent Themes Common to Both Groups.

**Fig 3:**
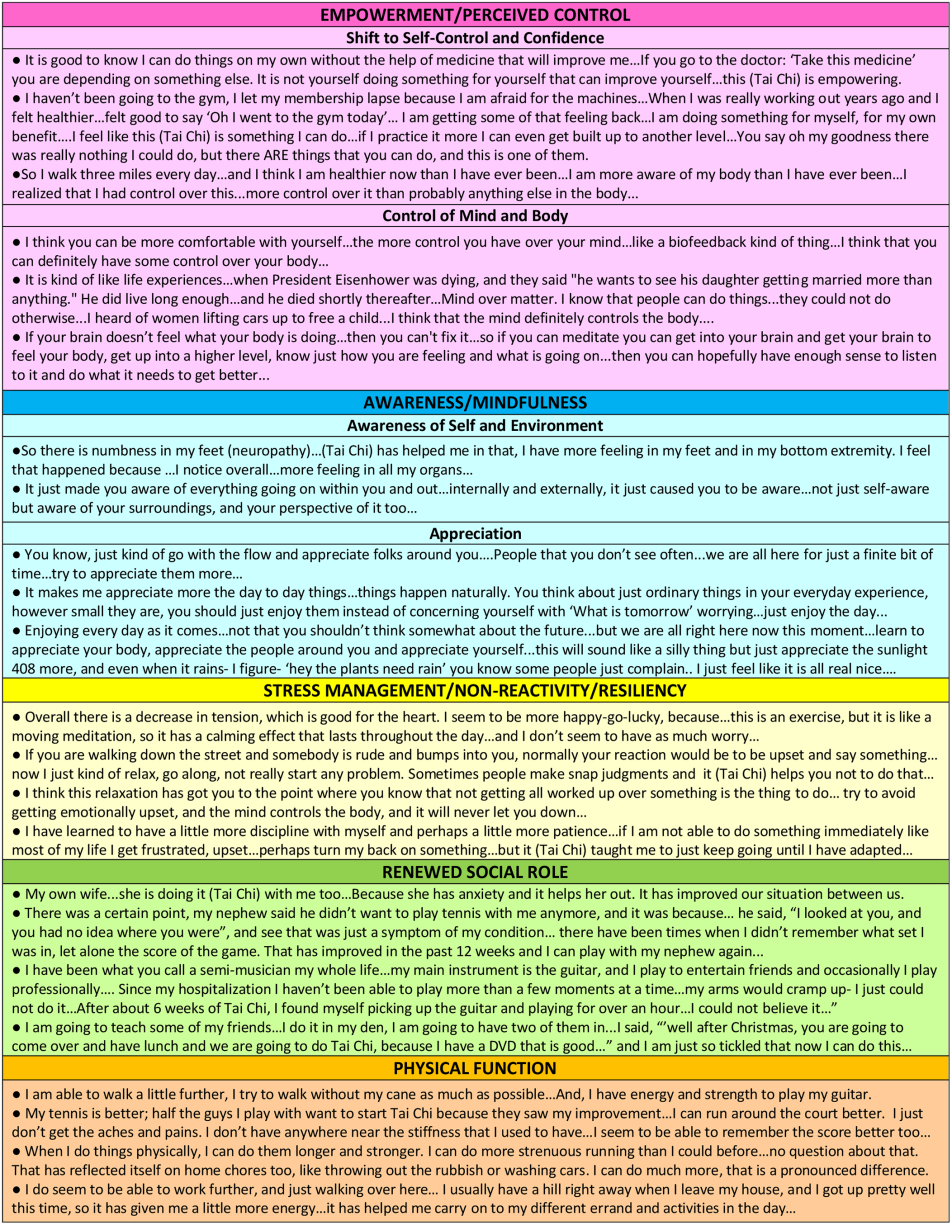
Representative Quotes Within Emergent Themes Unique in Tai Chi Participants.

Table 1 presents the characteristics of the sub-study population (N = 32). The mean age (±SD) of participants was 68 (±9) years, 59% were men, mean baseline ejection fraction was 29 (±7)%, mean NYHA class of 2. More than half of participants had a prior myocardial infarction. More than one-quarter to one-third of participants had either co-morbid anxiety, depression, or diabetes. About half the participants were married, and about a quarter were widowed, divorced, or separated. Fig 2 presents representative quotes from participants in both tai chi and education according to emergent themes that were common to both groups. Fig 3 presents representative quotes from tai chi participants according to emergent themes seen only in the exercise group. Multiple quotes within each category are from unique individuals.

**Table 1:** Baseline characteristics of study population.

*Characteristic*	Tai Chi (N = 17) N(%)*	Education (N = 15) N(%)*	Total (N = 32)** N(%)*	Total (N = 100)*** N(%)*
**Age, mean years (SD)**	71(10)	66(7)	68(9)	67(11)
**Gender: Men**	8(47)	11(73)	19(59)	64(64)
**Race/Ethnicity**				
White	17(100)	12(80)	29(91)	86(86)
Black	0(0)	3(20)	3(9)	11(11)
Other	0(0)	0(0)	0(0)	3(3)
**Annual household income**				
<25,000	2(11)	4(27)	6(19)	26(26)
25,000–49,999	3(17)	4(27)	7(22)	18(18)
50,000 or more	8(47)	6(40)	14(44)	42(42)
Decline	4(25)	1(6)	5(15)	14(14)
**Marital Status**				
Married	8(47)	11(73)	19(59)	59(59)
Widowed	4(23)	0(0)	4(12)	16(16)
Divorced/Separated	1(5)	1(6)	2(6)	13(13)
Single	4(23)	3(20)	7(22)	11(11)
**Primary Insurance**				
Private	9(52)	8(54)	17(53)	43(43)
Medicare	7(41)	5(33)	12(37)	44(44)
Medicaid	1(5)	2(13)	3(9)	12(12)
Other	0(0)	0(0)	0(0)	1(1)
**NYHA Class, mean (SD)**	2.0(0.6)	1.7(0.5)	1.9(0.6)	1.9(0.6)
**Baseline EF, % (SD)**	27(7)	31(7)	29(7)	29(7)
**Co-morbidities**				
MI	8(47)	10(66)	18(56)	58(58)
COPD	1(5)	3(20)	4(13)	18(18)
Diabetes	3(17)	6(40)	9(28)	37(37)
Depression	6(35)	4(26)	10(31)	30(30)
Anxiety	6(35)	3(20)	9(28)	30(30)
Severe arthritis	1(5)	3(20)	4(13)	14(14)
Charlson Co-morbidity Index	3(2)	3(2)	3(2)	3(2)

*N(%) unless otherwise noted

** Qualitative sub-study total sample

***Parent study total sample

### Emergent Themes in Both Groups

#### Self-efficacy (for diet and physical activity)

One significant theme that emerged from qualitative interviews in both groups was self-efficacy. Self-efficacy is a psychological construct based on Social Cognitive Theory, which describes the interaction between behavioral, personal, and environmental factors in health and chronic disease. The theory proposes that patients’ confidence in their ability to perform specific health behaviors influences their engagement in and actual performance of those behaviors, which in turn influences health outcomes.[28–29]

Both tai chi and education groups described improved self-efficacy to perform specific health behaviors such as physical activity and dietary modification. For example, participants felt particularly able to monitor and manage their salt intake both at home and outside of the home, and importantly, in challenging social situations. Both groups also mentioned that the program brought physical activity to the forefront and made them feel better able to implement exercise. Those in tai chi specifically described how the nature of tai chi contributed to their confidence in performing exercise, particularly in comparison to other conventional exercise options. In contrast, in previously published quantitative data in the parent study, we showed signifiant changes in Cardiac Exercise Self Efficacy in tai chi which were not detected in the education group, and we did not include an instrument to measure self-efficacy for diet behaviors.[15]

#### Social support

Participants in both groups expressed a strong sense of community and group social support. They described a sense of belonging and comfort being part of a group of patients that had a unique shared understanding that others without heart failure might not have. Participants described companionship and comraderie, and both groups mentioned gaining inspiration or motivation from others’ successes.

There was also a strong sense that being able to share common, difficult experiences was beneficial. Participants found inherent value in hearing other’s illness perspective, particularly negative experiences, functional limitations, or other challenging situations. Some expressed a certain comfort in having suffered through similar adversities, or in realizing their condition may not be as bad as others.

Although both groups, shared common, difficult experiences related to their conditions, in the education group, there were more expressions of commiseration, while in the tai chi group, many participants expressed an additional level of compassion and empathy towards the other members they thought less fortunate. These themes were not captured in previous quantitative measures.

### Emergent Themes Specific to Tai Chi

#### Empowerment/perceived control

Although both groups expressed increases in self-efficacy related to diet and exercise, the tai chi group described a more global empowerment and perceived control. Empowerment in health is defined as an interactive process between the self and the environment by which an individual gains greater control over decisions and actions that affect his or her health.[30–31] This has also been described as perceived control, which has been used more recently in the HF literature.[32–33] Empowerment and perceived control are broader concepts that encompass the related psychological constructs of, not only self-efficacy (described above), but also locus of control. Those exhibiting more internal (vs. external) locus of control feel that the control over situations and experiences that affect one’s life comes from within (and not outside of) the individual.[34]

Participants in the tai chi group described being in control over their own health and feeling empowered to do things they thought would have a beneficial impact on their condition. In many participants, there was a described shift of power from the doctor to the patient, from reliance on pharmaceuticals to self-care, and otherwise a shift from relative helplessness to a sense of control and confidence. Some described a control over their bodies through a newly cultivated awareness and power of their mind.

#### Awareness/mindfulness

Participants in tai chi described a heightened or new sense of awareness. Many described the cultivation of self-awareness that they practiced as part of their tai chi exercises. Participants described awareness of the breath, of their own bodily sensations, body signals, and symptoms, and how this allowed the forming of a new connection to themselves. There was also an increased awareness of others and of the environment. With increased awareness, many participants described an associated appreciation for family, friends, and social networks. Some expressed appreciation for nature, the world around them, and life in general.

#### Stress management and non-reactivity/resiliency

Participants described a general relaxation and calmness that was cultivated through the tai chi exercise that was helpful to relieve stress. They also described a new ability to act more resiliently with less automaticity in generating the usual negative emotions and actions to a given difficult situation or stressor.

#### Renewed social role

Participants described improvement with relationships and a return to prior family or other social roles that were previously meaningful, but at some point lost. For many, there was a renewal or redefining of important social roles in a new way that offered new purpose or meaning.

Each of the above themes unique to the tai chi group are new areas not captured in the previously analyzed quantitative results. We did, however, report significant improvements in disease specific quality of life (Minnesotal Living with Heart Failure Questionnaire) and mood (Profile of Mood States),[15] which align with the qualitatively reported psychosocial themes.

#### Physical function

In addition to the many psychosocial and emotional themes that emerged, many physical benefits were noted. These included improved strength, energy, flexibility, balance, decreased pain and stiffness, and overall endurance. In comparison to our previously reported quantitative measures, while we did not detect differential changes in exercise capacity (peak oxygen uptake and 6-minute walk) between groups, physical activity (moderate-intensity activity outside the study class measured by the CHAMPS questionnaire) was significantly increased in tai chi participants at 12 weeks.[15] Other qualitatively reported physical benefits were not quantitiatively assessed or measured but support additional areas of future investigation.

## Discussion

Important themes emerged (e.g., self-efficacy, social support, empowerment, stress resiliency, awareness and renewed social role), some of which mirror our previously reported quantitative results, and others providing further discernment and potential explanatory understanding.[15] In particular, social support and self-efficacy emerged as major themes in both groups. However, the tai chi program offered a broader empowerment and global perceived control that was not seen in the education group beyond self-efficacy for diet and exercise. These findings may offer additional insight into the mechanisms of tai chi and suggest possible relationships between self-efficacy, social support, empowerment/perceived control, and quality of life in patients with HF.

We previously reported that exercise self-efficacy improved significantly with tai chi over the education group.[15] While the questionnaire we employed quantitatively (Cardiac Exercise Self-Efficacy Instrument) measured self-efficacy for exercise, in our qualitative sub-study, we found other examples of self-efficacy, including confidence in diet-related behavior change. Open-ended qualitative questionning allowed us to find emergent themes and detect changes in self-efficacy in the education group that we were not equipped to see with the quantitative measure. In the tai chi narratives, we also found evidence of internal locus of control and overall empowerment. We previously reported that quality of life (as measured by the Minnesotal Living with HF questionnaire), an important primary outcome of the larger study, significantly improved with TC as compared to education. Our qualitative data supports the notion that overall self-efficacy, locus of control, and empowerment may be mediators of quality of life, and this may explain some of the differential improvement in HF quality of life with tai chi. Moving forward, these suggested relationships can be tested quantitatively.

Clinical research investigating patient empowerment and perceived control is most prevalent in the diabetes literature. This literature has recognized that exploring the meaning of these related constructs can have tremendous impact on patients’ diabetes self-management behaviors and help identify at-risk patients for further intervention.[35] Heart failure is similar to diabetes—in both conditions there exists a dramatic decrease in sense of control (less internal locus of control). This is also seen with cardiac patients post-myocardial infarction, and breast cancer patients after mastectomy.[10,33] In addition, similar to diabetes, treatment for HF requires lifelong adherence to medical self care regimens. Better understanding patient-centered factors such as perceived control and self-efficacy may allow development of relevant strategies to improve patient outcomes.

The literature on perceived control, specifically in HF, is sparse. One study by Rydlewska found that increasing perceived control is associated with increased self-efficacy, decreased depressive symptoms, and decreased BNP.[36] In other cardiac populations, less perceived control prior to an acute coronary event predicts in-hospital complications, and after acute myocardial infarction or revascularization, level of perceived control is more predictive of psychosocial recovery than physical factors such as NYHA functional class.[32–33] The relationship between perceived control and QOL in HF is also a new area of exploration. Initial work by Banerjee reported an unclear association between perceived control and quality of life after considering demographic, clinical, and psychological factors, with the suggestion that psychological status was more important.[37] More recently, other studies have found that perceived control is independently associated with QOL in HF, even after controlling for depressive symptoms and functional class.[38] These findings suggest that the relationship between these measures is likely complex.

In addition, we found that social support was profoundly important to both groups. Prior literature has suggested an association between self-efficacy, social support, depression and treatment adherence in HF.[39] Based on traditional social cognitive theory, two important facilitators of self-efficacy include ‘modeling’ and ‘social persuasion’.[28] Modeling describes how seeing others succeed can contribute to one’s own confidence to succeed and ‘social persuasion’ embodies the positive effects of group social support and peer encouragement. Both of these sub-themes within social support (Motivation from others, shared understanding, comraderie; Shared common difficult experience) emerged in our transcripts. Mirroring others’ findings,[18,40] particularly with tai chi, participants felt part of a unique, shared, transformative experience, and the environment fostered connection, support, understanding of illness, and shared success. This describes some ways that tai chi may have contributed to self-efficacy and perhaps broader empowerment and perceived control. We also noted a subtle difference in how participants in the two groups responded to the group experience (Commiseration vs. Empathy). While the education group shared common struggles and difficulties, as did the TC group, the latter expressed an additional level of empathy and compassion. This raises the possibility that certain aspects of social support may be more influential than others in affecting overall empowerment. For example, support from peers, caregivers, or other social networks that further provides empathy and compassion may be particularly empowering and this may further mediate quality of life.

Living with heart failure is often characterized by increased stress, feelings of helplessness compromised physical function, and social role dysfunction.[2] In this analysis, we found that tai chi offered patients additional strategies, such as mindfulness/awareness, stress resiliency and appreciation of self and others that may directly address these issues, and the return to or renewal of a prior social role was an expressed benefit by some in the tai chi group. Each of these may further contribute to increase in self-efficacy and global empowerment. For example, stress resiliency allows one to stop prior to an automatic anger reaction in response to a stressor, and instead take a milder, less emotionally charged course, which offers an increased sense of control over one’s thoughts, feelings, and actions, which may in turn affect overall perceived quality of life.

Within the field of contemplative science, there is a robust literature describing the effects of mindfulness and awareness. Central to these themes is the construct of interoception, the sense of receiving and appraising signals within the body. Interoception is said to be critical for one’s sense of embodiment, motivation and well-being. By definition, this creates both presence- one’s connection to the moment (mindfulness), and agency- one’s ability to effect change (related to empowerment), and both are critical in determining one’s sense of well-being.[41] Recently, there is acknowledgement that the cultivation of interoceptive, proprioceptive, and kinesthetic awareness is at the core of many movement based mind-body therapies like tai chi.[42] Related to this kinesthetic awareness, some studies have reported increased tactile acuity in tai chi practitioners, as well as increase ankle and knee proprioception in older adults after tai chi.[43–45]

With tai chi, improvements in physical function were described including improved strength, energy, flexibility, balance, decreased pain and stiffness, and overall endurance which supports prior literature.[18,39] Another important facilitator of self-efficacy according to traditional theory is prior success. With the experience of improved physical function in multiple domains, future improvements and successes are more accessible and perceived control improves. While our qualitative analysis supported an increase in endurance with tai chi that was not detected in our quantitiative measures of peak oxygen uptake and 6-minute walk,[15] other investigations of mind-body therapies have suggested impacts on exercise capacity.[46–47]

## Conclusion

With the recognition that heart failure is a progressively debilitating disease, this qualitative analysis supports recent emphasis on patient-centered outcomes and living well, where perceived functional status, psychosocial and emotional wellness are just as important indicators of patient success as measured oxygen consumption, cardiac events, and mortality.

Future studies of mind-body exercise might further examine the relationship between self-efficacy, locus of control, empowerment and quality of life, better understand these measures as potential mediators of effect, and propose ways to tailor interventions or target at-risk populations with HF as well other chronic disease.

## Supporting Information

S1 File Protocol. (DOC)

S2 File CONSORT Checklist. (DOC)
