# Supplementary material for: The Impact of Tai Chi Exercise on Self-Efficacy, Social Support, and Empowerment in Heart Failure: Insights from a Qualitative Sub-Study from a Randomized Controlled Trial
Source: PLoS One. 2016 May 13;11(5):e0154678. doi: 10.1371/journal.pone.0154678 (PMC4866692; doi:10.1371/journal.pone.0154678)
Supplement: S1 File — (DOC) [file pone.0154678.s001.doc]

## DESCRIPTION OF RESEARCH PROTOCOL (PARENT STUDY)

## 1. Study Design

We propose a prospective, randomized controlled trial assessing the efficacy of Tai Chi mind-body movement therapy as an adjunct to standard care in patients with chronic stable heart failure. We will compare a twelve-week Tai Chi program to an education attention control. This study will be named “NEXT-HEART Study”, which is short for **N**ew **Ex**ercise **T**ai Chi and **H**eart **E**ducation: **a R**andomized **T**rial. Measurements at baseline, 6 and 12 weeks will include quality-of-life and behavioral/psychosocial assessments, six-minute walk test, and neurohormone/cytokine blood tests. Metabolic bicycle stress test will be done at baseline and 12 weeks only. In addition, we will conduct a brief follow-up phone call at 6 months to assess current health status, quality of life using the Minnesota Living with Heart Failure Questionnaire, use of healthcare (numbers of hospitalizations and emergency department visits since the 12-week interview), and practice of Tai Chi and other exercise.

Figure 1: Study Flow Diagram

## Subject Selection

The sample will be identified from the ambulatory clinics at three main institutions: Beth Israel Deaconess Medical Center (BIDMC), Brigham and Women’s Hospital (BWH), and Massachusetts General Hospital (MGH). In addition to the two advanced HF specialty clinics (BIDMC and BWH) utilized in our pilot, we will also be recruiting from the primary care and cardiology clinics at each of the hospitals and affiliated community practices. The HF clinics combined see approximately 4000 patients per year. For our pilot study, we were able to enroll 30 patients over 1 year from two clinics with very minimal recruiting resources.

Hebrew Senior Life (HSL): We will also recruit eligible subjects from Hebrew Senior Life in Roslindale and two community housing facilities associated with HSL: Jack Satter House in Revere and the Simon Fireman Community in Randolph.

Most sites are concentrated in the same 3-block radius on the Harvard Longwood Medical Campus. The MGH is located approximately 3 miles from Longwood with a shuttle bus available between sites every 15 minutes. In addition, we will advertise the study via newspapers and flyers.

As HF is primarily a disease of the elderly, we expect to have mainly a population >60 years of age. We do not expect to have many patients below the age of 30, although this is theoretically possible with entities such as peripartum cardiomyopathy. Based upon our pilot data, we expect that more than 50% of patients will be ≥65 years of age, and over 80% will be ≥55. Patients will be excluded if they cannot safely complete the necessary testing measurements (such as exercise stress testing). We will recruit both men and women, as well as participants of all races and ethnicities.

**Subject Incentives**

Patients will be paid $200 for participating ($100 at 6 weeks and $100 at study end). The study may also facilitate transportation to and from study-related visits if logistical or financial constraints impair the patients’ ability to participate. Subjects recruited from Hebrew Senior Life Facilities will be paid $250 for their participation ($125 at 6 weeks and $125 at study end). Because classes will be held on site at Hebrew Senior Life (HSL) Facilities and we do not have to pay for parking, we are able to increase the incentives for subjects participating at these facilities.

At the end of the study, we will offer patients randomized to the education control group Tai Chi classes at no costs for 3 months as a courtesy. For the Tai Chi group, we will offer subjects the opportunity to continue taking Tai Chi. Tai Chi subjects may attend one additional 3-month Tai Chi program (free of charge) within 6 months of completing intervention class, but will be required to pay their own parking expenses.

### Eligibility Criteria

We will recruit patients using the following inclusion and exclusion criteria.

**Inclusion Criteria:**

- Adult patients with chronic HF
- Left ventricular ejection fraction ≤40%, by echocardiogram or by left ventriculogram in the past 2 years*
- Stable medical regimen, defined as no major changes in medication in the past 3 months
- New York Heart Association Class I, II, or III

**Exclusion Criteria:**

- Unstable angina or myocardial infarction in the past 3 months
- Major cardiac surgery within the past 3 months
- History of cardiac arrest in the past 6 months
- History of cardiac resynchronization therapy (CRT) in the past 3 months
- Unstable serious ventricular arrhythmias
- Unstable structural valvular disease
- Current participation in a conventional cardiac rehabilitation program
- Diagnosis of peripartum cardiomyopathy within prior 6 months
- Inability to perform a bicycle stress test
- Lower extremity amputation, or other inability to ambulate due to conditions other than HF
- Severe cognitive dysfunction precluding informed consent and understanding of Tai Chi concept (MMSE ≤ 24)
- Inability to speak English
- Current regular practice of Tai Chi
- Participant randomized to Tai Chi in Tai Chi Pilot Study or current participant in other trial

*For patients who appear otherwise eligible for the study, but who have not had their ejection fraction (EF) measured in the past 2 years will be asked to undergo a limited echocardiogram at MGH to measure their current EF and confirm study eligibility. This will include patients such as those with a chronic stable low EF whose medical providers believe that the patient’s EF or condition has not changed enough to warrant an echocardiogram for clinical purposes. For such patients, informed consent will take place either prior to or during their echocardiography visit following the study procedures outlined in Procedures for Informed Consent.

*We will not exclude patients on the basis of their religious preferences or practices.*

Taking into account the general demographics of the 3 study institutions, we anticipate the following profile of patients with respect to women and minorities.

| **GENDER** | **MINORITIES** |
| --- | --- |
| White Women 53 | Whites 105 |
| African American Women 15 | African American 30 |
| All Women 75 | All minorities 45 |
| Percent female 50% | Percent minority 30% |

### Intervention

We will utilize the same Tai Chi protocol that we developed, refined, and successfully employed in our pilot study. This intervention, broadly based on a Tai Chi program developed by Wolf and colleagues for use in trials with elderly patients^17^ emphasizes essential Tai Chi movements that are both easily comprehensible and can be done repetitively in a flowing manner. The five chosen Tai Chi movements––*‘raising the power’*, *‘withdraw and push’*, *'grasp the sparrow's tail'*, *‘brush knee twist step’*, and *‘wave hand like clouds’* –– are based on the traditional Cheng Man-Ch’ing’s Yang-style short form. In addition to these five formal movements, the intervention includes a complementary set of traditional Tai Chi warm-up exercises and a 2 minute cool-down period with breathing exercises. These focus on loosening up the physical body, incorporating mindfulness and imagery into movement, increasing awareness of breathing and promoting overall relaxation of body and mind. This intervention was developed by Dr. Peter Wayne who has more than 25 years of Tai Chi experience.

Two senior Tai Chi students and certified graduates of a 2.5 year instructor training program developed by Dr. Wayne will administer the Tai Chi intervention under Dr. Wayne’s direction. Some instructors taught classes for the pilot study and have already gained experience working with a frail cardiac population and appreciate the unique set of challenges this population may bring. Prior to the start of the trial, Dr. Wayne will conduct a series of trainings to review the protocol. Video analysis of mock group sessions will be used to ensure consistent application of the protocol.

Tai Chi classes will be conducted twice weekly for one hour each for 3 months (identical to the intervention used in our pilot study). Many prior Tai Chi trials have demonstrated clinical changes using a similar-length intervention.

Classes will be held at BIDMC, MGH Revere Community Health Associates, and other community-based facilities. These facilities are located in outpatient clinical space and thus, are fully equipped with medical emergency equipment and have immediate access to medical personnel. A nurse or study staff member certified in basic life support will be present at all classes. Chairs will be provided for seated warm-up exercises and resting, as well as for stability as needed when performing standing exercises. Throughout the 12-week program, the nurse or BLS-certified study staff member will maintain a log to document class attendance, and who completed the class with or without sitting down.

Hebrew Senior Life: Classes will be held in fitness centers located at Hebrew Senior Life Roslindale Campus, Jack Satter House, and Simon Fireman Community.

To facilitate logistical and transportation issues, we will offer classes at a variety of times, including weekday, weekend, and evening sessions. Of note, HF educational materials provided to the education control group (described below) will also be given to patients in the intervention group. Materials will be handed out weekly and briefly presented (3-5 minutes) by the study nurse or BLS-certified study staff member prior to the start of Tai Chi class.

| Table 1. Details of Proposed Tai Chi Intervention | | |
| --- | --- | --- |
| Week | Activities | Approximate Duration (min) |
| 1 | Introductory Session: Overview of Program  1. Description of class format  2. Demonstration of Tai Chi form  3. Expectations of participants  4. Tai Chi principles, philosophies   1. Participation in Warm-up Exercises 2. Cool-down (breathing) | 5  10  5  10  30  2 |
| 2-5 | Warm-up Exercises (Repeated during all sessions)   1. Standing:   a) “Drumming the body”  b) “Swinging to connect kidney and lungs”  c) “Washing the body with qi”  d) Standing meditation (w/ breathing)   1. Sitting:   a) Neck/Shoulder stretches  b) Arm/Leg stretches  c) Sitting meditation (w/ breathing)  Total Warm-up time  Tai Chi Movements   1. “Raising the Power” 2. “Withdraw and Push’   Cool-down (Breathing) | 6  3  3  3  6  3  6  30  5-10  5 per side  2 min |
| 6-9 | (Warm-up and Movements 1-2)  3. “Grasp Sparrows Tail”  4. “Brush Knee Twist Step”  Cool-down (Breathing) | 5 per side  5 per side  2 min |
| 10-12 | (Warm-up and Movements 1-4)  5. “Wave Hands Like Clouds”  Cool-down (Breathing) | 5-10  2 min |

### Attention Control Comparison Group

The standard comparison arm in parallel conventional exercise trials has historically been usual care. Currently underway is the HF-ACTION trial, a large multi-site NIH-funded study comparing usual care plus a conventional exercise program to usual care alone. Although we felt this design was reasonable for our pilot investigations, we recognize its inherent limitations. One important criticism for a usual care comparison group is that it does not properly control for the group social interaction that is present in our class-based intervention. We will address this issue in this proposal with a time-matched attention control.

Patients in the control group will attend education/social group sessions twice weekly. Classes will be structured as shown in Table 2. The first fifteen minutes of education will be led by the study nurse and follow the content of a series of pamphlets designed by the Heart Failure Society of America (HFSA) (we will purchase from [www.hfsa.org](http://www.hfsa.org/)). Additional information about blood cholesterol will be used from the National Heart, Lung and Blood Institute (NHLBI). These “Education Modules” are self-contained and written in easy-to-understand language. .

Module topics include:

1. Taking control of your heart failure
2. How to follow a low-sodium diet
3. Heart failure medicines
4. Self-care and dealing with HF symptoms
5. Exercise and activity
6. Managing feelings about heart failure
7. Tips for family and friends
8. Lifestyle changes
9. Advanced directives
10. Heart rhythm problems
11. New heart failure treatments
12. High blood cholesterol.

Topics will be distributed over the 12-week study period. Although more intensive educational interventions with HF nurses as part of a comprehensive multi-disciplinary management team have shown to be beneficial, there are actually few data on the effects of minimal education interventions in HF. While generally believed to be helpful, we do not expect these education pamphlets to have a major impact on our primary endpoints.

The remaining 45 minutes will consist of semi-structured group time moderated by the study nurse. Patients will be asked to discuss a given topic related to the HF module. We do not intend for this time to be used for medical consultation. The nurse will have explicit instructions to serve as moderator only and not to make any recommendations regarding an individual’s treatment. Throughout the 12-week program, the nurse will maintain a log to document class attendance

| Table 2. Details of Proposed Education Attention Control | | |
| --- | --- | --- |
| Week | Activities | Approximate Duration (min) |
| 1 | Introductory Session: Overview of Program  1. Description of class format  2. Description of Education Modules  3. Expectations of participants  Semi-structured Social Group | 5  5  5  45 |
| 2-12 | Presentation of HF Education Module content  Semi-structured Social Group | 15  45 |

As noted previously, patients in the intervention group will also receive the same 11 educational pamphlets, handed out weekly at Tai Chi class.

### Measurement Protocols

Patients will be tested at three time points: baseline, 6 weeks, and 12 weeks. Patient interview, questionnaires, blood tests, and six-minute walk will be conducted at all three time points. Cardiac tests (bicycle metabolic stress test) will be done only at baseline and 12 weeks. Tests will be coordinated to maximize patients’ convenience. BIDMC and BWH are within close walking distance on the Longwood Medical Campus. Taxi vouchers will be available should patients require the short cab ride. A free shuttle bus is available every 15 minutes between the BWH and MGH. As mentioned previously, we will conduct a brief follow-up phone call at 6 months to assess current health status, quality of life using the Minnesota Living with Heart Failure Questionnaire, use of healthcare (numbers of hospitalizations and emergency department visits since the 12-week interview), and practice of Tai Chi and other exercise.

**Exercise Capacity and Functional Status**

### Six Minute Walk Test

The six minute walk test is a standardized assessment that measures the distance walked in meters down a corridor at a comfortable pace. The test will be done early in the visit on each of three occasions, at least 2 hours before the bicycle ergometer test. Patients will be read standardized, scripted instructions by a research assistant who is blinded to treatment arm. The assessor will stand in silence at one end of the straight, uncluttered corridor and will inform the patient when there are 3 minutes and 1 minute before the end of the test. Patients will be allowed to stop as often as they need if they fatigue. The six-minute walk test has been shown to be an independent predictor of HF prognosis and survival in pharmacologic trials.

### Timed Get-Up and Go Test

We will conduct the timed “Timed Get-Up and-Go", which is a reliable and valid test for quantifying functional mobility that may also be useful in following clinical change over time. The "timed get up and go test" requires patients to stand up from a chair, walk a short distance (3 meters, ~10 feet) at a comfortable pace, turn around, return, and sit down again. This test will be timed by the blinded research assistant. The patient will repeat the test and the times will be averaged. The test is quick, requires no special equipment or training, and is easily included as part of the routine medical examination. The timed score has been shown to be reliable and to correlate well with log-transformed scores on the Berg Balance Scale (r = -0.81), gait speed (r = -0.61) and Barthel Index of ADL (r = -0.78). The “timed get up and go test” will be performed at baseline, 6-weeks and 12-weeks.

### Peak Oxygen Uptake on Bicycle Metabolic Stress Test

Bicycle metabolic stress tests will be performed either at the Cardiac Testing Center at Brigham and Women’s Hospital or at Beth Israel Deaconess Medical Center and will be paid for by NIH grant award R01 AT002454. Technicians and physicians performing tests will be blinded to study group. Patients will perform a symptom-limited exercise test using a bicycle ramp protocol to determine peak VO_2_. Testing will be done on an electronically calibrated upright bicycle, with expired gas analysis under continuous electrocardiographic monitoring. Blood pressure will be taken at 3-minute intervals and just prior to stopping exercise. Participants will be encouraged to exercise to exhaustion. Perceived exertion will be measured using the 1-10 Borg Scale. Respiratory gas analysis will be performed on a breath-by-breath basis using a Sensormedic metabolic cart. Peak values will be averaged from the final 20 seconds of the test. Peak VO_2_ has been shown to correlate with cardiac output and skeletal muscle blood flow and has been an important criterion to assess when patients should undergo cardiac transplantation. Study investigators will send a copy of the test results to the attending physician with consent of the study subject.

## Quality of Life (QOL) and Psychosocial Functioning

*Patient interviews will be conducted at baseline, 6 weeks, and 12 weeks. All questionnaires will be administered by a research assistant who is blinded to the patient’s study group.* We will pilot test the survey instruments on 5 patients to determine participant burden.

To capture health-related QOL, we will use a disease-specific questionnaire, the Minnesota Living with Heart Failure (MLHF) Questionnaire and a generic Medical Outcomes Survey Short Form (SF-36) health status survey. Comparisons of these instruments in heart failure suggest that a more comprehensive assessment is gained by using multiple instruments. In addition, we will use the Profile of Mood States assessment (POMS) to more specifically capture mood and emotional status.

### Disease-Specific QOL Measure

The main QOL measure (and one of the primary endpoints that are accounted for in the power calculation) will be the disease-specific MLHF. This validated instrument is the most-commonly employed health status measure in HF. It consists of 21 items covering physical, psychological, and socioeconomic dimensions (e.g. swelling in the ankles, difficulty climbing stairs, fatigue, feeling depressed, spending money for HF). The score range is 0 to 105, with a lower number denoting better QOL. Prior studies have reported that a score of seven indicates some degree of impaired quality of life and that an improvement of five points represents a clinically significant change.

### Emotional Status/Mood

The POMS is a well-validated instrument for assessing emotional states that are transient and expected to respond to clinical intervention. It has been widely used to assess the effects of exercise interventions. The instrument consists of 65 single-word items rated on a 5-point scale to indicate recent mood in 6 dimensions: tension/anxiety, depression/dejection, anger/hostility, vigor/activity, fatigue/inertia, and confusion/bewilderment. A decreased total mood disturbance score denotes an improved emotional state. Test-reliability coefficients are reported to range from 0.65 to 0.74.^80^ Studies of Tai Chi have reported improvement in mood, decrease in anxiety, and enhancement in vigor as measured by the POMS scale.

### Self-Efficacy

The Cardiac Exercise Self-Efficacy Instrument (CESEI) is a validated 16-item scale that assesses patient’s confidence to perform certain physical exercises on a 5-point scale (no confidence to very confident). Score range is 16-80, with a higher number denoting increased self-efficacy. Stability and internal consistency estimates are about 0.9.

## Neurohormonal Status

*Blood tests will be conducted at baseline, 6 weeks, and 12 weeks for all patients. All blood tests will be obtained and analyzed by the BIDMC General Clinical Research Center. However, test results from the various blood tests will not be provided to the attending physician for two reasons. First, the General Clinical Research Center is not a CLIA certified lab and is not results are not allowed to be used for clinical purposes. In addition, blood tests are not run in “real time”; instead they will be run in batches at the end of the study period. Patients eligible for this study are typically a highly monitored patient population and these tests are not meant to substitute for routine medical care.*

BNP samples will be analyzed on whole blood collected in EDTA using a commercially available Biosite Triage BNP Test point-of-service meter (fluorescence immunoassay). Serum BNP >100 pg/ml supports a diagnosis of symptomatic heart failure. TNF-α and c-reactive protein will be determined using commercial ELISA kits.

## Autonomic Tone

### Catecholamines

Plasma norepinephrine, epinephrine, and dopamine will be measured by high performance liquid chromotography/electrochemical detector. Resting catecholamine samples will be drawn on ice in heparinized tubes after 20 minutes of lying quiet in a supine position with intravenous catheter in place. Analyses for norepinephrine, epinephrine, and dopamine will be performed using high-performance liquid chromatography/electrochemical detector. Norepinephrine level has been identified as one of the strongest predictors of HF progression, functional status, and death. In the Studies of Left Ventricular Dysfunction(SOLVD), norepinephrine levels above the median value were twice as likely to be associated with death from HF.^33^

## Medical Utilization and Collection of Cost Data

Medical record, interview data, and automated utilization data will be used to document patients’ use of health care during the study period. We will collect information on visits to healthcare providers, telephone contact with health care providers, use of prescription and over the counter medications, use of radiology and laboratory tests, hospitalizations, and other invasive or non-invasive cardiac procedures (e.g., cardiac catheterizations, cardioversion, pacemaker implantation). We will track both insurance payments and out-of-pocket expenses.

We will compare the health care resources used by patients randomized to education attention control to resources use by patients randomized to Tai Chi. Information will be obtained through patient self-report. Studies that have primarily relied on patient interview have found that the average error in self-reported utilization is small.^97^ However, for those patients referred from the BIDMC, we will also utilize the hospital database and online medical record system that tracks office visits, telephone contacts, hospitalizations, emergency room visits, radiology and laboratory tests, procedures, and prescription medications. This will allow us to check self-reported utilization by comparing self-report data with that captured through the hospital database. We will not track these electronic data at any other site since database systems of those hospitals are not fully integrated online, and thus, would not yield comparable information.^97^ A standardized Medicare Fee Schedule will be used to assign an estimate of costs in dollars to hospitalizations and visits to physicians and nurses. The fee schedule also will assign cost estimates to prescription medications, lab and radiology tests, and cardiac procedures. Costs of over the counter medications will be estimated based on wholesale costs and average national dispensing costs.

## Other Data Collection

We will track patient attendance at study classes for both groups and compliance with home Tai Chi practice for the Tai Chi group. The Study Nurse will also monitor patients blood pressure and heart rate immediately before and after class at baseline, 6, and 12 weeks.

To keep track of patients’ level of physical activity outside of Tai Chi sessions, we will use the CHAMPS Physical Activity Questionnaire.^98^

## Qualitative Interviews

In a subset of 30-35 patients, we will also perform semi-structured qualitative exit interviews to further explore areas not captured in our standardized quantitative instruments. Particularly, we will be interested in patients’ candid assessments (positive, negative, and neutral) of various aspects of the Tai Chi intervention. These open-ended questions may yield additional insights into understanding mechanism of a mind-body exercise program and to further explore the relationships between beliefs, attitudes, and expectations. Interviews in the control group participants will also give comparative information regarding the education-attention control. One-hour exit interviews will be conducted at the end of the study by a study nurse or investigator and will be audiotaped. These audiotapes will be transcribed verbatim to provide transcripts of the interviews, which will be used for qualitative analyses. The transcripts will be reviewed in conjunction with the taped interview to ensure accuracy and the data will be analyzed. The audiotapes and transcripts will be kept in a locked cabinet in the research assistant’s locked office until all transcription and reviews have been performed. The audiotape will not be duplicated, distributed, or used for any purpose other than those described in this study. Once the investigators are satisfied that the data accurately reflects the taped interview, the audiotapes will be destroyed.

## 2. STATISTICAL CONSIDERATIONS

### Sample Size Justification

We will enroll a total of 100 patients, with 50 patients in each group. We estimate that 75 of patients will be enrolled from BIDMC and 25 patients from other study sites (eg. BWH and MGH).

The sample size is based on power calculations using data from our pilot study. For these calculations, we assumed a 1) 10% loss of efficiency because we will use non-parametric tests and 2) 20% dropout rate. Although all patients enrolled in the pilot study completed that study, we did have a few missing data points. Therefore we felt it would be prudent to anticipate a larger dropout rate given prior experiences with HF patients.

Since there are three primary endpoints (change in six-minute walk, peak VO_2_, MLHF score), we will apply a Bonferroni adjustment to preserve the 5% level of significance. Based on pilot data, the standard deviation (SD) for change in walk test distance=317 feet; SD for change in peak VO_2_=2.1 ml/kg/min; and SD for change in MLHF score=17 points. Accounting for the above, the sample of 50 patients per group provides 80% power to detect a peak VO_2_ difference of 1.5 ml/kg/min between groups. We will have >90% power to detect a walk test difference of 240 ft and 83% power to detect MLHF score difference of 12.5 between groups, each of which is half the magnitude of difference observed in the pilot study. These differences represent clinically meaningful changes in functional capacity and quality of life.

### Statistical Analysis and Power

Prior to analyzing the outcomes, we will compare the randomized groups with respect to baseline demographic, functional, and clinical variables as well as known prognostic factors (such as baseline NYHA class and baseline peak VO_2_) using chi-square and Fisher’s exact tests. Although we do not expect any differences with proper randomization, any significant imbalances (p<.05) will be adjusted for in the statistical analyses. All analyses will be performed on an intention-to-treat basis.

## Data Management And Analysis Plan

*All data management and statistical analyses will be performed at BIDMC under the direction of the senior biostatistician. Analyses will be performed primarily using SAS statistical software.*

To protect confidentiality, all data will be stored a locked file cabinet located in a secured office in the Division of General Medicine and Primary Care at BIDMC staffed by research personnel. All computers and data files will be password protected. All data processed will be in aggregate form and every study subject will be assigned a unique identification number. The Master List linking subject names to their ID numbers will be maintained in a locked cabinet in the Project Director’s office.

All data, such as eligibility criteria, questionnaires, laboratory values, and results of cardiac testing will be entered electronically and verified for accuracy by a second research assistant who is not blinded to study group. At regular intervals, collected data will be checked for completeness and appropriate ranges of numerical data. All primary data including data collected in paper form will be stored in locked file cabinets in an office staffed by research personnel from this study. Back-up files of all computerized databases will be maintained in the Division of General Medicine and Primary Care under the direction of the biostatistician. Data will be rigorously managed by the biostatistician and project manager to confirm data completeness and quality.

### Analysis Plan for Primary Specific Aims

To assess the adequacy of randomization, we will use Fisher’s Exact tests (categorical variables, e.g., sex, NYHA Class) and Wilcoxon rank-sum tests (continuous variables, e.g., age) to detect any differences in baseline characteristics between treatment groups.

Our three primary endpoints are change in exercise capacity and quality of life from baseline to 12 weeks using the six-minute walk, peak VO_2_ and MLHF score. Because we will recruit patients from 3 institutions, we will formally examine whether there is a site effect by performing Kruskall-Wallis tests to assess whether changes from baseline to 12 weeks differ by site for each outcome. In the event that we observe a site effect, we will proceed stratifying our analyses by site using van Elteren’s test. A priori, however, we do not expect to observe a site effect for a number of reasons, including the 3 institutions are academic tertiary care centers located in the same catchment area, they have similar patient profiles, and they apply the same standard of cardiac care by following ACC/AHA guideline. If no site effect is observed, we will proceed with our analyses by performing two-sample non-parametric Wilcoxon rank-sum tests to compare the distribution of change from baseline to 12 weeks between treatment and control groups for each outcome measure.

### Analysis Plan for Secondary Aims

For neurohormonal markers: BNP, TNF-alpha, c-reactive protein, and norepinephrine, two-sample Wilcoxon rank-sum tests will also be used to compare distribution of change from baseline to 12 weeks between intervention and control groups.

Scores on other quality of life, mood, and exercise self-efficacy assessments will be analyzed using similar tests: Wilcoxon rank-sum, comparing the change from baseline to 12 weeks between treatment and control groups. In addition, we will use linear regression models using primary study outcomes (change in six-minute walk, peak VO2, and MLHF score) as dependent variables and scores on these psychosocial assessments as predictors to examine whether clinical outcome is associated with quality-of-life, mood, or self-efficacy. Separate models will be developed for each primary outcome. In the Tai Chi intervention group, we will also describe patient attendance at classes and compliance with home practice and determine whether compliance is associated with either baseline exercise self-efficacy or a positive change in exercise self-efficacy.

Medical utilization costs of the two groups will be compared using Wilcoxon rank-sum tests, examining cost components (outpatient visits, telephone contacts, hospitalizations, radiology and laboratory tests, procedures, and medications) separately and in total. We will also use linear regression models to adjust for baseline patient characteristics, such as sociodemographic variables (race) or clinical variables (co-morbidities and NYHA class).

### Additional Analyses

Other descriptive analyses: Clinical data obtained through patient interview (as well as medical record/automated utilization data used to track medical resource use for the cost analysis) will be analyzed using descriptive statistics. Particularly, we are interested in medication changes such as diuretic or beta-blocker dosage, unforeseen cardiac procedures such as pacemaker placement, cardiac hospitalization and ER visits that may be informative if there are clinically significant differences between the groups. In addition, patient report of physical activity outside of study sessions (as measured by the CHAMPS instrument) will be analyzed and presented descriptively.

Qualitative exit interview: Qualitative patient exit interviews will be professionally transcribed from audiotapes and coded using an inductive approach informed by grounded theory methods. We will identify passages in the transcripts that represent common themes or content categories relating to positive, negative, and neutral aspects of patients’ experiences with both Tai Chi intervention and education-attention control. Data will be analyzed and presented descriptively according to content categories.

### Data Safety and Monitoring Board

Data safety and monitoring will be carried out by the independent Data Safety and Monitoring Board (DSMB) established for this trial. The DSMB will meet at least once prior to the start of patient recruitment and twice a year during the 3-year period in which patients are involved in Tai Chi sessions. Our first meeting of the DSMB was March 4, 2005 in preparation for subject recruitment beginning in April 2005. The DSMB is comprised of three members: a senior biostatistician, a cardiologist, and tai chi expert. With the guidance of the DSMB, we will develop a protocol for identifying, monitoring, and reporting adverse events. The focus of the first DSMB meeting will be to review and approve the protocol; specify the list of expected adverse events and serious adverse events to be monitored throughout the trial; approve the timeline and mechanisms for reporting AEs; and specify the reports/data requests for future DSMB meetings. In addition, we will report adverse events according to the following policies (see table below).

| **Adverse Event Grading Scale and Reporting Protocol** | | | |
| --- | --- | --- | --- |
| **Severity** | **Include in Annual report to CCI** | **Written Report to CCI, GCRC RSSO, Parners’ and NESA’s IRBs**  **(within 5 working days)** | **Written Report to NCCAM (within 5 working days)** |
| Mild, Expected | **✓** |  |  |
| Mild, Unexpected | **✓** |  |  |
| Moderate, Expected | **✓** | **✓** |  |
| Moderate, Unexpected | **✓** | **✓** |  |
| Serious, Expected | **✓** | **✓** | **✓** |

All study personnel will be given a copy of the protocol for reporting adverse events once it is finalized. We will designate a “safety monitor” who will be responsible for compiling information on adverse events and reporting them to the Principal Investigator and other appropriate individuals (depending on whether the AE is serious or not and expected or unexpected). The safety monitor will be either the study nurse or physician associated with the study. The study nurse or BLS-certified study staff member will attend and monitor subjects for potential adverse events during the Tai Chi class. In addition, we will monitor adverse events identified by the DSMB during the follow-up interviews at 6 and 12 weeks. **Serious unexpected** adverse events will be reported within 24 hours after the study team learns of the SAE and will follow up with a written report to the CCI and GCRC Research Subject Safety Office (RSSO) at BIDMC as well as appropriate affiliate IRBs within 5 working days.

As described above, the initial meeting of the DSMB was held March 4, 2005 in preparation for subject recruitment beginning in April 2005. This initial meeting identified a preliminary list of the expected and unexpected adverse events that will be tracked throughout the study. The DSMB reviewed and approved the following rule to suspend patient accrual.

We will be enrolling 100 chronically ill patients with New York Heart Association (NYHA) Class I, II, and III heart failure who are at an increased risk of dying. The DSMB will review all deaths that occur during the 12-week study period. However, because mortality is not an endpoint of this study, it is inadequately powered to detect a difference in mortality between the treatment groups (i.e., Tai Chi versus education attention control). Because we will be following subjects for only a 12-week period, it is unlikely that there will be a sufficient number of deaths to achieve statistical significance.

Therefore, we will adopt the following rule: For subjects with NYHA Class I and II heart failure, if the overall mortality rate exceeds 10% based on at least 5 deaths, we will suspend patient accrual until the DSMB conducts a full review. For subjects with NYHA Class III heart failure, if the overall mortality rate exceeds 25% based on at least 5 deaths, the study will suspend patient accrual until the DSMB conducts a full review. If either of these conditions is met, the DSMB will meet within two weeks to conduct a full review of all of the deaths. The review will consider whether the deaths were attributable to the study intervention by examining a) temporal relationship with the Tai Chi intervention; b) balance of deaths between treatment groups; and c) issues related to NYHA Class and severity of illness.

Finally, we will provide the CCI and GCRC RSSO with biannual reports submitted to the National Center for Complementary and Alternative Medicine (NCCAM) at NIH following each DSMB meeting and an annual summary of adverse events delineated by the categories described above.

## POSSIBLE BENEFITS

This study will increase our understanding of the potential benefits of a tailored 12-week Tai Chi program on functional and health-related quality of life in patients with chronic heart failure. Tai Chi has the potential to improve the physical functioning and quality of life for patients with living with congestive heart failure. This study will also enable us to better understand the mechanisms (physiologic and metabolic pathways and behavioral and psychosocial effects) underlying any benefits of Tai Chi. In addition, this study will provide data on the cost-effectiveness of Tai Chi as adjunctive therapy.

If such benefits are demonstrated, Tai Chi could represent a new adjunctive therapy for cardiac patients, which is not currently a part of standard care. Especially, in patients with heart failure who are difficult to engage in activity, a mind-body exercise form may be particularly appropriate and help to define optimal exercise protocols in this population. This study will also allow us to gain important knowledge regarding potential mechanisms of mind-body therapies that can be applied to other chronically ill populations where stress-related physiology plays an injurious role. Finally, we will offer all patients the benefit of participating in Tai Chi, regardless of their assign study group. For patients assigned to the education control group, we will offer free Tai Chi classes for 12 weeks after participating in the study.

## POSSIBLE RISKS AND ANALYSIS OF RISK/BENEFIT RATIO

Tai Chi is likely to be a relatively safe activity. We have found no reports of adverse effects in the literature and did not experience any adverse events during our pilot study in a heart failure population (identified using the same eligibility criteria). We will have the study nurse or BLS-certified study staff member present at all Tai Chi classes. These classes will be held in facilities that have immediate access to hospital emergency equipment and personnel.

Other risks of the study are those arising from assessment. We realize that our population is fragile and at risk for cardiac events. With the six-minute walk or bicycle stress test, the possibility of arrhythmias, hyper-or hypotension, severe shortness of breath, or ischemia exists. Theoretically, decompensation of cardiac function could occur with any excessive exertion in this population. Falls are also possible with the six-minute walk. Patients undergoing bicycle stress testing will be fully monitored and supervised by a cardiologist at all times with emergency equipment on hand and at least 2 ACLS-trained staff persons nearby. In general, seated bicycle tests are usually better tolerated than treadmill tests. In our experience, these tests are well tolerated and complications are rare.

## RECRUITMENT AND CONSENT PROCEDURES

## Procedures for Patient Identification and Enrollment

To identify potential patients for enrollment, we will use on-site screening of patients at clinic visits; regular screening of potential patients using the electronic medical records; referral from physicians and other providers; newspaper advertisements; and flyers. All recruitment procedures will follow HIPAA guidelines.

Patients will be recruited from the primary care, heart failure and general cardiology clinics at the three participating institutions: Beth Israel Deaconess Medical Center, Brigham and Women’s Hospital, Massachusetts General Hospital, and affiliated community practices. At these institutions, we will solicit referrals from providers (physicians and nurse practitioners) practicing in these clinics and leave informational brochures and flyers in patient areas. The research nurse coordinator and research assistants will maintain constant contact with providers through email communication, monthly staff meetings, and regular site visits. We will pre-screen the electronic medical record to identify potential patients with an ejection fraction of ≤40%. With the provider’s permission, we will pre-screen patients with appointments from clinic appointment logs for study eligibility. We will also obtain from each provider whether they would like to us to ascertain approval to approach each eligible subject individually. For these providers, we will notify them of each eligible patient and request their permission to approach the patient to invite participation. We will also ask whether another physician is involved in the patient’s care who should be contacted for permission. However, some providers request that we only contact them once an eligible patient has expressed strong interest in study enrollment. For these providers, we will notify them of each eligible patient who expresses interest in study enrollment and ask whether there is another provider involved in the patient’s care who should be contacted for permission. At this juncture, providers have the opportunity to decline study participation for any reason.

We will approach patients at their clinic visit (after approval from their provider), call patients, and/or send letters introducing the study and inviting patients to participate. When inviting patients to participate over the phone, we will use a prepared phone script for the initial contact. This phone script will serve as a guide for study staff to provide patients with information about the study and its procedures in as consistent manner as possible. The letter will provide information about the study and a number to call for persons who want additional information about the study. For patients who are interested in additional information, the research assistant or nurse will explain the protocol, answer questions, determine eligibility, and discuss informed consent. If patients are interested but not eligible, they will be referred back to their primary cardiologist for other exercise or stress reduction options. Those interested and eligible may be scheduled for a baseline visit.

In addition, we will leave informational brochures and hang flyers at the Marino Center for Integrative Medicine in Cambridge, MA and in and around the New England Medical Center (NEMC) to enhance our recruitment efforts. The Marino Center, a full service medical clinic offering integrative conventional and complementary therapies, provides care to patients interested in complementary therapies while NEMC provides services to a large Asian population. We will post flyers at BIDMC and the Longwood Medical Area. To further expand recruitment, we will place an advertisement in local newspapers.

For persons interested in the study who come to us via these recruitment methods, a member of the study staff will talk to the person over the phone, explain the protocol, answer questions, and discuss informed consent. For those patients who remain interested, the study staff will pre–screen the patient for heart failure and study eligibility. Study staff will be guided by the phone script to ask patients if we can contact their primary physician and/or cardiologist to ascertain medical information to confirm that they are eligible to participate. The name and address of their doctor(s) will be recorded on the form for screening patients over the phone. We will tell patients that we will send them a consent form which gives us permission to contact their primary physician and/or cardiologist. A follow-up letter will be sent to potentially eligible subjects along with an eligibility form for their primary physician and/or cardiologist to complete and the consent form to contact their doctor(s). In some instances, we may send the eligibility form to physicians directly if the patient agrees and in other instances patients may prefer to gather their medical records and have our clinical staff abstract eligibility information. Once interested patients are determined to be eligible they may be scheduled for a baseline visit. For patients who do not qualify for the study, we will ask them if they would like us to keep their name on file in case they become eligible in the future or for other studies.

**Recruitment at Hebrew Senior Life Facilities**

We will recruit eligible patients from the Hebrew Senior Life in Roslindale and residents of the Jack Satter House and Simon Fireman Community. Recruitment at the Hebrew Senior Life facilities in Roslindale will rely on direct physician referral, searches of online medical records and “patient keeper” to identify potentially eligible subjects using ICD-9 diagnosis codes for heart failure, medication and problem lists. We will then determine final eligibility by further chart review and/or physician correspondence. To recruit eligible subjects from the community housing facilities in Revere and Randolph, we will send an informational letter and brochure to all residents with a number to call for anyone interested, post flyers at the facilities, and seek direct referrals from the housing facility’s on-site physicians. Those who call will undergo further phone screening to determine likelihood of eligibility using the procedures for phone screening and determining eligibility outline above.

## Procedures for obtaining Informed Consent

Written informed consent will be obtained by the research nurse coordinator, the research assistant, or study investigator prior to or during this initial visit. Patients will be asked to participate in a study comparing Tai Chi to a HF education intervention. Patients who are unable to complete a baseline bicycle stress test (e.g., have unexpected ischemic changes or life-threatening arrhythmias during testing) will be excluded, although this circumstance is expected to be rare. Randomization will therefore take place after baseline testing. Treatment assignments will be generated by a permuted blocks method with randomly varying block size and sealed in numbered, opaque envelopes. Randomization will be stratified based on NYHA heart failure class (class I/II versus class III). Once final eligibility is confirmed, the research nurse coordinator or unblinded research assistant will open the next envelope in sequence and the patient will be informed of assignment.

## 
